# Predictors of extubation failure and prolonged mechanical ventilation among patients with intracerebral hemorrhage after surgery

**DOI:** 10.1186/s12931-023-02638-5

**Published:** 2024-01-04

**Authors:** Ue-Cheung Ho, Chia-Jung Hsieh, Hsueh-Yi Lu, Abel Po-Hao Huang, Lu-Ting Kuo

**Affiliations:** 1 Division of Neurosurgery, Department of Surgery, National Taiwan University Hospital, Yunlin Branch No. 579, Sec. 2, Yunlin Rd, Yunlin, 640 Taiwan; 2 Graduate Institute of Clinical Medicine, College of Medicine, National Taiwan University, Taipei, 100 Taiwan; 3 Division of Neurosurgery, Department of Surgery, National Taiwan University Hospital, Taipei, 100 Taiwan; 4 Department of Industrial Engineering and Management, National Yunlin University of Science and Technology, Yunlin, 640 Taiwan; 5 Institute of Polymer Science and Engineering, National Taiwan University, Taipei, 100 Taiwan

**Keywords:** Extubation failure, Intracerebral hemorrhage, Mechanical ventilation, Intubation

## Abstract

**Background:**

Spontaneous intracerebral hemorrhage (ICH) is a condition associated with high mortality and morbidity. Survivors may require prolonged intubation with mechanical ventilation (MV). The aim of this study was to analyze the predictors of extubation failure and prolonged MV in patients who undergo surgical evacuation.

**Methods:**

This retrospective study was conducted on adult patients with ICH who underwent MV for at least 48 h and survived > 14 days after surgery. The demographics, clinical characteristics, laboratory tests, and Glasgow Coma Scale score were analyzed.

**Results:**

A total of 134 patients with ICH were included in the study. The average age of the patients was 60.34 ± 15.59 years, and 79.9% (n = 107) were extubated after satisfying the weaning parameters. Extubation failure occurred in 11.2% (n = 12) and prolonged MV in 48.5% (n = 65) patients. Multivariable regression analysis revealed that a white blood cell count > 10,000/mm^3^ at the time of extubation was an independent predictor of reintubation. Meanwhile, age and initial Glasgow Coma Scale scores were predictors of prolonged MV.

**Conclusions:**

This study provided the first comprehensive characterization and analysis of the predictors of extubation failure and prolonged MV in patients with ICH after surgery. Knowledge of potential predictors is essential to improve the strategies for early initiation of adequate treatment and prognosis assessment in the early stages of the disease.

## Background

Spontaneous intracerebral hemorrhage (ICH) is a condition associated with high morbidity and mortality, accounting for 20–40% of strokes [1–3]. Predictors of ICH outcome include age, Glasgow Coma Scale (GCS) score, ICH location, volume, and other imaging findings such as the presence of a midline shift and intraventricular extension [4, 5].

Approximately 30% of patients with ICH require intubation during hospitalization [6, 7]. Prolonged intubation, defined as mechanical ventilation (MV) use, for > 14 days, is required in 10–23% of intubated patients with ICH [8–12]. In addition, extubation failure, defined as the inability to sustain spontaneous breathing without MV, is prevalent in approximately 15–43% of these patients [7, 13–15]. Both extubation failure and prolonged MV are associated with longer intensive care unit (ICU) stays, higher mortality, and other complications [16, 17].

Some patients with ICH require surgical treatment, but the possible risk factors for extubation failure and prolonged MV remain unclear in these patients. This study aimed to identify the predictors of extubation failure and prolonged MV in patients with ICH who underwent surgical evacuation of ICH.

## Methods

### Study design

This retrospective study included patients with ICH admitted to the ICU after surgery who were endotracheally intubated for at least 48 h with MV and survived > 14 days. All patients were followed up for at least 1 month until they underwent spontaneous breathing trials (SBTs) and passed the weaning parameters before extubation. Patients who were unable to meet the weaning criteria within 2 weeks following the surgery or required re-intubation within 48 h of extubation were categorized as having prolonged mechanical ventilation. Patients who were unable to maintain spontaneous breathing and required re-intubation within 48 h of extubation were categorized as extubation failure. This study was conducted at the National Taiwan University Hospital in accordance with the applicable local regulations and the Declaration of Helsinki. It was approved by the institutional review board of the same institution (IRB number: 201611058RINA). As patients were comatose, written informed consent was obtained from their caregivers.

### Population

All patients were admitted to the National Taiwan University Hospital between May 2015 and December 2019. A total of 208 patients (age > 18 years) with more than 30 mL of unilateral ICH at the basal ganglia, thalamic hemorrhage with or without intraventricular hemorrhage causing hydrocephalus, lobar, or cerebellar hemorrhage with a hematoma diameter > 3 cm or causing hydrocephalus, and who underwent ICH surgery were included. The following patients were excluded: those with pre-existing brain diseases (meningitis, stroke, or brain tumor), substance abuse disorders (alcohol or illicit drugs), history of lung surgery, use of MV for < 48 h after ICH surgery, or survival time of < 1 month after ICH. Patients who signed a do-not-resuscitate (DNR) order were also excluded. We collected baseline information, including patient demographics, medical history of hypertension or diabetes mellitus, laboratory data on admission and before extubation, imaging data, and diagnosis-related information.

### Surgical procedures and assessments

Prehospital management was based on the standards of the Taiwan Society of Emergency Medicine. Upon arrival at the emergency department, triage nurses assessed the patients’ medical condition. A physician evaluated the GCS score and pupillary reactions to light. A brain computed tomography (CT) scan was performed immediately. The manual *ABC/2* formula was employed in this study to estimate the volume of the ICH. In this formula, ‘*A*’ represents the diameter of the largest cross-sectional area of the hematoma by CT, ‘*B*’ denotes the vertical diameter of this same cross-sectional area (that is, the diameter 90° to *A*), and ‘*C*’ is the approximate number of CT slices with hemorrhage multiplied by the slice thickness which quantifies the thickness of the hematoma layer.

All patients who met the criteria underwent surgical treatment for ICH, including endoscopy-assisted hematoma evacuation and intraparenchymal placement of a fiber-optic intracranial pressure (ICP) monitor (Camino Laboratories, San Diego, CA, USA; model 110-4BT), craniotomy for hematoma evacuation, or placement of external ventricular drains (EVD), based on the GCS score, pupillary examination, CT findings, age, hematoma volume and location, and neurological deterioration, such as GCS decrease or abnormal papillary response to light.

### Postoperative care

All patients were admitted to the ICU immediately after surgery. Postoperative management included MV, fluid resuscitation, use of antiepileptic medications (valproic acid or levetiracetam), antibiotic prophylaxis for at least 3 days, and early enteral nutrition using a nasogastric or orogastric tube. Standard postoperative monitoring procedures at the ICU included continuous invasive measurements using an arterial catheter, blood oxygen saturation using a pulse oximeter, end-tidal CO_2_ concentration, and blood sugar level. Hourly neurological assessments were performed, including GCS score, pupil size, and reaction to light. A 2-hour ICP > 20 mmHg assessed by the monitor or external ventricular drain during the first 3 days after surgery was considered an indication for brain CT scanning. Otherwise, a scan was performed on postoperative day 3.

### Measurement of weaning parameters and institution of protocolized weaning program

After the patients’ condition stabilized post-ICH surgery without any elevation in the ICP, a gradual reduction of MV support was initiated for the transition from controlled to spontaneous ventilation. A protocolized weaning program was implemented to prepare patients for extubation, which includes an assessment of their cough reflex. Once the patients could tolerate the reduced applied airway pressure support of 5–10 cmH_2_O, an SBT was performed using a T-tube (T-piece) to assess the ability to breathe spontaneously. Simultaneously, weaning parameters, including maximal inspiratory pressure (PiMax), maximum expiratory pressure (PeMax), respiratory rate (RR), rapid shallow breathing index (RSBI), minute ventilation (VE), and tidal volume (VT) were measured by a respiratory therapist using a standardized protocol [9]. In our hospital, the cutoff points of weaning parameters were − 30 cmH_2_O for PiMax, 30 cmH_2_O for PeMax, RSBI < 105, RR < 30 breaths/min, VE < 10 L/min, and VT > 5 mL/kg. Following successful completion of the T-piece trial, eligibility for extubation were assessed by evaluating weaning parameters and assessing the patient’s cough reflex one day prior to the planned extubation. Once patients successfully met most of weaning criteria and demonstrated the ability to cough effectively, extubation was performed in the morning, regardless of whether it was a weekend or a week day.

If the patient did not require reintubation after monitoring for 48 h, the weaning process was regarded as successful. We implemented a standardized post-extubation respiratory support protocol that included interventions such as high-flow nasal cannula, chest physiotherapy, high-frequency chest wall oscillation, and airway secretion clearance when deemed necessary. In addition, a post-extubation swallowing screening assessment was administered as a precautionary measure to mitigate the risk of re-intubation.

For patients undergoing EVD placement, we conducted the weaning of EVD in parallel with the MV weaning process. However, in cases where patients exhibited persistent hydrocephalus throughout their treatment course, we opted to retain the EVD for cerebrospinal fluid (CSF) diversion to avert neurological deterioration. The weaning program remained unaltered for these patients, and extubation was conducted provided that they had satisfactorily met the majority of the weaning criteria and demonstrated effective coughing capabilities. Patients in need of continuous CSF diversion received either lumbar drain insertion or ventriculoperitoneal shunt placement, as determined by the treating surgeon’s clinical judgment.

### Statistical analyses

Continuous variables were presented as mean ± standard deviation in the descriptive analyses, whereas categorical and binary variables were presented as frequencies (n) and percentages (%). The chi-squared test and Student’s *t*-test were used to compare outcomes between patient subgroups for categorical and continuous data, respectively. Univariate analyses were performed using logistic regression analysis. Multivariable logistic regression with stepwise selection was performed to identify the independent predictors of extubation failure and prolonged intubation. Missing data were not included in the analysis. Statistical significance was set at *P* < 0.05. Data were analyzed using SPSS 26 for Windows (SPSS, Inc., Chicago, IL, USA).

## Results

### Demographic data

**Fig. 1:**
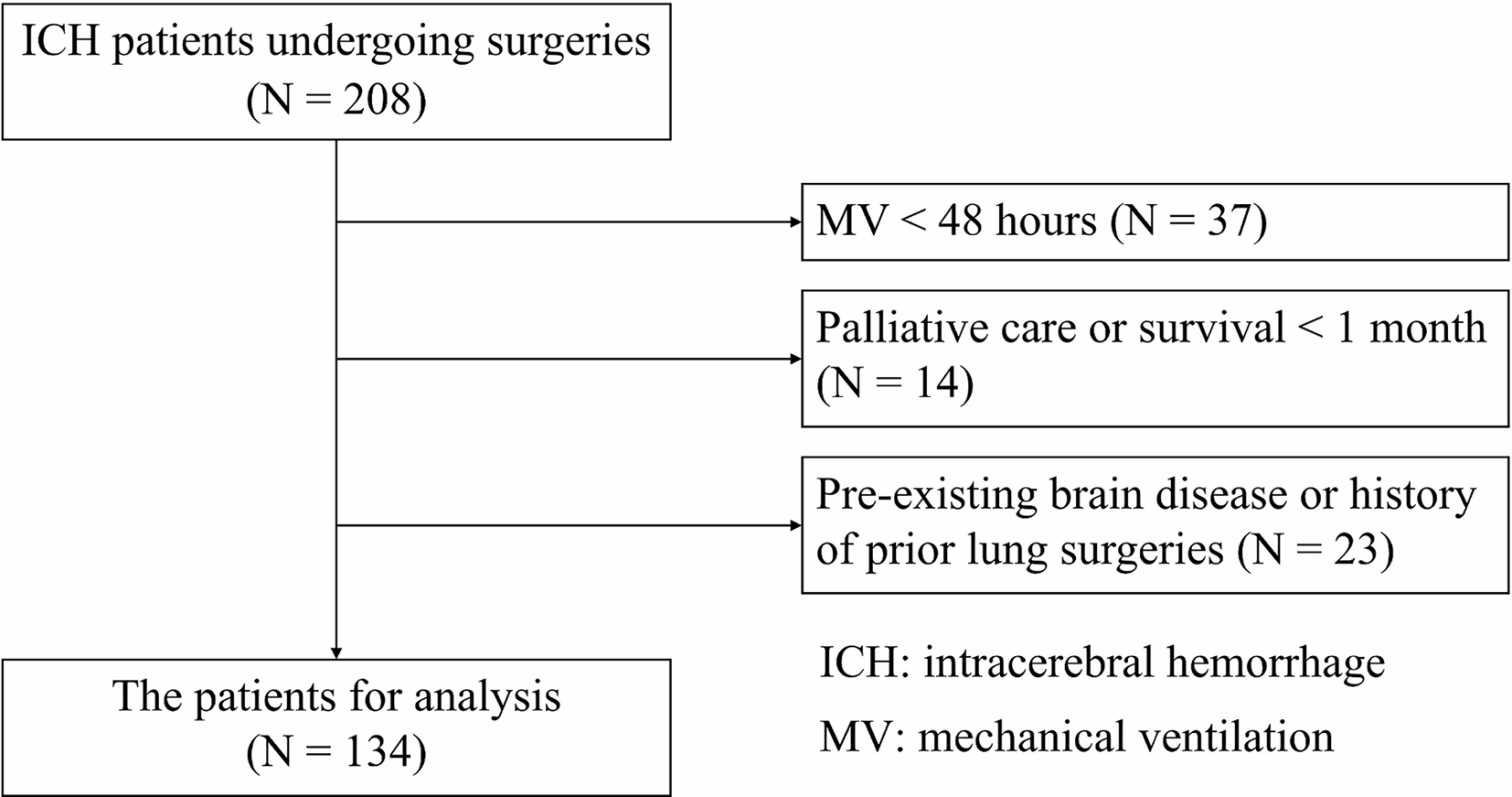
Flow chart of patient enrollment

Of the total 208 patients enrolled, we excluded 37 patients who required mechanical ventilation for less than 48 h following ICH surgery, 14 patients who had a documented DNR and were deemed eligible for palliative care, with a projected survival period of less than 1-month post-ICH, and 23 patients with pre-existing brain disease or a history of prior lung surgeries. Finally, 134 patients with ICH who underwent surgery and survived for ≥ 2 weeks and underwent MV ≥ 48 h postoperatively, were included in the study (Fig. 1). The mean age was 60.34 ± 15.59 years, with male predominance (63.4%, n = 85). In terms of ICH location, deep-seated was most common (51.5%, n = 69), followed by lobar (38.1%, n = 51), and cerebellar (10.4%, n = 14). The overall median score of GCS was 9. The mean hematoma volume was 38.30 ± 24.01 mL. Patients were mechanically ventilated for a mean duration of 9.9 ± 8.5 (range: 2–23) days before extubation (Table 1).

**Table 1:** Characteristics of the patient cohort with intracerebral hemorrhage (n = 134)

Variable	Mean ± standard deviation or n (%) or median with interquartile range
Age (years)	60.34 ± 15.59
Sex, male	85 (63.4)
Weight (kg)	66.78 ± 15.28
Diabetes mellitus	36 (26.9)
Hypertension	88 (65.7)
Coronary artery disease	14 (10.4)
Hemodialysis	5 (3.7)
**Intracerebral hemorrhage location**	
Lobar	51 (38.1)
Deep-seated	69 (51.5)
Cerebellar	14 (10.4)
Intracerebral hemorrhage volume (mL)	38.30 ± 24.01
**Surgical procedure**	
Endoscopy-assisted procedure	50 (37.3)
Craniotomy procedure	84 (62.7)
External ventricular drain (EVD) placement	37 (27.6)
Initial eye-opening response	3 (2)
Initial motor response	5 (2)
Initial verbal response	1 (2)
Initial Glasgow Coma Scale score	9 (5)
Initial white blood cell count (/mm^3^)	13.54 ± 19.73
Initial white blood cell count > 10,000/mm^3^	79 (59.0)
Initial blood glucose (mg/dL)	157.34 ± 60.38
Initial blood glucose > 100 mg/dL	127 (94.8)
Initial blood glucose > 120 mg/dL	100 (74.6)

### Factors associated with extubation failure

During the postoperative intensive care, 107 patients (79.9%) were weaned off MV after passing the SBT. Most (88.8%, n = 95) had successful extubation without reintubation and a mean MV duration of 10.13 (range: 2–23) days. Patients with extubation failure had a mean MV duration of 8.25 (range: 2–20) days before the first trial. In the extubation failure group, all patients required re-intubation due to their inability to maintain their airway pressure and exhibited limited expectoration ability following extubation. The mean duration between extubation and reintubation was 1.5 days. None of these patients were re-intubated due to deteriorating neurological status. Table 2 compares the demographic and clinical data and the weaning parameters between those with successful and failed extubations.

**Table 2:** Univariate analysis of the factors associated with extubation failure (n = 107)

Variable	Successful extubation (n = 95)	Failed extubation (n = 12)	*P*-value
	Mean ± standard deviation or n (%) or median with interquartile range		
Age (years)	59.61 ± 14.51	61.08 ± 19.00	0.75
Sex, male	62 (65.3)	6 (50.0)	0.30
Weight (kg)	66.88 ± 1.60	69.42 ± 16.50	0.60
Diabetes mellitus	24 (25.3)	3 (25.0)	0.98
Hypertension	62 (65.3)	8 (66.7)	0.92
Coronary artery disease	10 (10.5)	3 (25.0)	0.15
Hemodialysis	4 (4.2)	1 (8.3)	0.52
**Intracerebral hemorrhage location**			0.88
Lobar	35 (36.8)	4 (33.3)	
Deep-seated	49 (51.6)	6 (50.0)	
Cerebellar	11 (11.6)	2 (16.7)	
Intracerebral hemorrhage volume (mL)	37.94 ± 24.03	46.91 ± 24.69	0.89
**Surgical procedure**			0.65
Endoscopy-assisted procedure	32 (33.7)	4 (33.3)	
Craniotomy procedure	63 (66.3)	8 (66.7)	
Initial eye-opening response	3 (2)	3 (2)	0.54
Initial motor response	5 (2)	5 (1)	0.91
Initial verbal response	1 (3)	1 (2.5)	0.88
Initial Glasgow Coma Scale score	10 (4.75)	9 (5.5)	0.73
Initial white blood cell count (/mm^3^)	11.47 ± 5.23	12.14 ± 4.05	0.67
Initial white blood cell count > 10,000/mm^3^	55 (57.9)	8 (66.7)	0.56
Initial blood glucose (mg/dL)	156.40 ± 64.57	143.83 ± 35.76	0.51
Initial blood glucose > 100 mg/dL	89 (93.7)	11 (91.7)	0.79
Initial blood glucose > 120 mg/dL	71 (74.7)	8 (66.7)	0.55
Pre-extubation eye-opening response	3 (1)	3 (1.75)	0.48
Pre-extubation motor response	6 (1)	6 (1)	0.53
Pre-extubation verbal response	1 (0)	1 (0)	0.62
Pre-extubation Glasgow Coma Scale score	10 (2)	10 (2.75)	0.77
Pre-extubation white blood cell count (/mm^3^)	10.62 ± 3.60	12.39 ± 3.16	0.11
Pre-extubation white blood cell count > 10,000/mm^3^	48 (50.5)	10 (83.3)	0.03*
Pre-extubation hemoglobin (g/dL)	10.78 ± 1.82	10.50 ± 1.53	0.61
Pre-extubation sodium (mmol/L)	136.47 ± 4.35	136.21 ± 3.14	0.84
**Pre-extubation weaning parameters**			
Respiratory rate	21.98 ± 5.03	25.00 ± 4.75	0.05
Respiratory rate > 30 breaths/min	6 (6.3)	3 (25.0)	0.03*
Maximal inspiratory pressure (cmH_2_O)	-43.82 ± 12.22	-38.50 ± 10.77	0.15
Maximal expiratory pressure (cmH_2_O)	49.08 ± 14.87	41.42 ± 11.70	0.09
Maximal expiratory pressure > 46 cmH_2_O	59 (62.1)	3 (25.0)	0.02*
Tidal volume (mL/kg)	434.3 ± 241.21	377.93 ± 53.235	0.42
Minute ventilation (L/min)	8.74 ± 2.84	9.42 ± 2.16	0.42
Rapid shallow breathing index	60.45 ± 35.16	67.85 ± 16.89	0.48
Mean duration between extubation and reintubation (day)		1.5 ± 0.5	

* <0.05, ** <0.01 indicated the significance of bold values

No significant differences were observed in the initial GCS score, pre-extubation GCS score, or surgical procedures between the two groups. Similarly, there were no significant distinctions in hematoma location or volume between these groups either. Patients in the successfully extubated group were significantly less likely to have elevated white blood cell (WBC) count on extubation than those with extubation failure (50.5 vs. 83.3%, *P* = 0.03). Moreover, patients in the extubation failure group were more likely to have an RR of > 30 breaths/min than those in the successful group (25.0 vs. 6.3%, *P* = 0.03). Regarding PeMax of > 46 cmH_2_O, the difference was statistically significant between the groups (62.1 vs. 25.0%, *P* = 0.02).

### Factors associated with prolonged MV

Prolonged MV (≥ 14 days) was seen in 48.5% (n = 65) patients. Table 3 compares the demographic and clinical data and the weaning parameters between the two groups of MV: <14 days and ≥ 14 days.

**Table 3:** Univariate analysis of the factors associated with prolonged mechanical ventilation (MV) (n = 134)

Variable	MV duration ≥ 14 days (n = 65)	MV duration < 14 days (n = 69)	*P*-value
	Mean ± standard deviation or n (%) or median with interquartile range		
Age (years)	63.29 ± 15.86	57.55 ± 14.87	0.03*
Age ≥ 65 years	36 (55.4)	20 (29.0)	0.00**
Sex, male	37 (56.9)	47 (68.1)	0.18
Weight (kg)	65.43 ± 13.09	68.08 ± 17.09	0.32
Diabetes mellitus	21 (32.3)	15 (21.7)	0.17
Hypertension	44 (67.7)	44 (63.8)	0.63
Coronary artery disease	8 (12.3)	6 (8.7)	0.49
Hemodialysis	4 (6.2)	1 (1.4)	0.15
**Intracerebral hemorrhage location**			0.85
Lobar	26 (40.0)	25 (36.2)	
Deep-seated	33 (50.8)	36 (52.2)	
Cerebellar	6 (9.2)	8 (11.6)	
Intracerebral hemorrhage volume (ml)	34.68 ± 24.93	39.66 ± 21.33	0.63
**Surgical procedure**			0.82
Endoscopy-assisted procedure	23 (35.4)	27 (39.1)	
Craniotomy procedure	42 (64.6)	42 (60.9)	
Initial eye-opening response	1 (2)	3 (2)	0.00**
Initial motor response	4 (2)	5 (1)	0.00**
Initial verbal response	1 (0)	2 (3)	0.00**
Initial Glasgow Coma Scale score	7 (3.25)	10 (5)	0.00**
Initial white blood cell count (/mm^3^)	15.82 ± 27.75	11.39 ± 5.13	0.19
Initial white blood cell count > 10,000/mm^3^	38 (58.5)	41 (59.4)	0.91
Initial blood glucose (mg/dL)	156.17 ± 45.33	158.44 ± 72.08	0.83
Initial blood glucose > 100 mg/dL	64 (98.5)	63 (91.3)	0.06
Initial blood glucose > 120 mg/dL	49 (75.4)	51 (73.9)	0.85

* <0.05, ** <0.01 indicated the significance of bold values

No significant differences were observed in the surgical procedures between the two groups. Similarly, there were no significant distinctions in hematoma location or volume between these groups either. Patients in the shorter MV duration group were significantly younger than those in the longer duration group (57.55 vs. 63.29 years, *P* = 0.03). They also had a significantly higher initial GCS score before surgery (10 vs. 7, *P* = 0.00). Each component of the initial GCS score was also higher in these patients (*P* = 0.01).

To identify the independent prognostic variables, multivariable regression analysis was performed using those variables with a *P* < 0.10 in the univariate analysis. WBC > 10,000/mm^3^ at the time of extubation was the only independent predictor of reintubation (Table 4). Age ≥ 65 years and initial GCS were identified as independent predictors of prolonged MV (Table 5). The AUC (area under the receiver operating characteristic curve) used to measure the performance of the prediction was 0.80 (sensitivity = 0.75 and specificity = 0.71) for extubation failure and 0.78 (sensitivity = 0.74 and specificity = 0.73) for prolonged mechanical ventilation.

**Table 4:** Multivariable analysis of the factors associated with extubation failure

Factors	OR	95% CI	*P*-value	Sensitivity	Specificity	AUC
Weaning WBC > 10,000/mm^3^	6.10	1.14–32.51	**0.03**	0.75	0.71	0.80
Respiratory rate	1.11	0.98–1.25	0.10			
PeMax	0.96	0.92–1.01	0.16			

*Abbreviations*: AUC, area under the receiver operating characteristic curve; CI, confidence interval; OR, odds ratio; PeMax, maximal expiratory pressure

**Table 5:** Multivariable analysis of the factors associated with prolonged mechanical ventilation

Factors	OR	95% CI	*P*-value	Sensitivity	Specificity	AUC
Age ≥ 65 years	3.28	1.50–7.20	0.00	0.74	0.73	0.78
Initial GCS	0.74	0.65–0.86	0.00			
Initial blood glucose	1.00	0.99–1.00	0.33			

*Abbreviations*: AUC, area under the receiver operating characteristic curve; CI, confidence interval; GCS, Glasgow Coma Scale; OR, odds ratio; PeMax, maximal expiratory pressure

## Discussion

To our knowledge, this study provides the first comprehensive characterization and analysis of the predictors of extubation failure and prolonged MV in patients who underwent surgery for ICH. The results of the multivariable analysis demonstrated that an elevated WBC count was associated with extubation failure, and age and initial GCS score were associated with prolonged MV.

Generally, intubation is indicated in patients with GCS scores < 8, significant respiratory distress, elective surgical intervention, and need for therapeutic hyperventilation [6, 18–20]. Weaning off MV is crucial in managing patients with ICH when their condition improves. Patients requiring MV are prone to skeletal muscle atrophy due to physical inactivity, and early weaning can improve this via physical rehabilitation. This process begins by assessing the patient’s readiness to wean based on an SBT [21], followed by the decision of extubation. Regarding respiratory physiology capacity, weaning parameters are used to assess readiness [22]. These commonly include PiMax, PeMax, VT, VE, RR, RSBI, airway resistance, and compliance [23, 24]. However, many patients with ICH and impaired consciousness have a decreased central respiratory drive and lose the ability to protect the airway and clear secretions. These patients may experience extubation failure even if they pass the weaning parameters because they are at high risk of airway occlusion and respiratory failure, necessitating intubation and MV for adequate airway protection and oxygenation [9, 15, 24, 25].

ICH location can be classified as deep-seated, lobar, or infratentorial. Deep-seated ICH is mostly restricted to the basal ganglia and thalamus, accounting for approximately two-thirds of spontaneous ICH [26, 27]. In comparison, lobar ICH may result from several diseases, especially those associated with cerebral amyloid angiopathy among the elderly population [28]. Infratentorial ICH includes brainstem and cerebellar hemorrhage. ICH can lead to brain damage due to local tissue destruction, thrombin-induced brain edema [29], hematoma lysis-associated edema [30], and increased ICP. These may result in long-lasting impairment of certain brain functions, which may require prolonged dependence on MV [10]. Prolonged MV is an important prognostic factor in several other diseases [31, 32]. Surgical hematoma drainage or evacuation has advantages in terms of reducing both, mass effect and lowering ICP. The choice of surgical approach depends on the location and size of ICH, ventricular involvement, clinical condition, the availability of surgical equipment in the hospital, and the surgeon’s technical expertise.

Traditional weaning parameters reflect the patient’s respiratory drive and ability to wean off MV; however, they cannot accurately predict the ability to protect the airway after extubation [33, 34]. Unsuccessful extubation is associated with increased hospital mortality, need for tracheostomy, longer MV duration, and longer ICU and hospital stay [16, 17]. Extubation failure has been reported to be associated with age, the severity of illness, use of intravenous sedation, GCS score, neurological impairment, duration of MV before extubation, secretion volume, and cough strength [17, 35, 36]; however, all these risk factors proposed in the literature have not been found to be consistently associated with extubation failure across the studies. Coplin et al. [37] reported that delayed extubation after passing the weaning criteria in brain-injured patients is more likely to lead to pneumonia, prolonged ICU and hospital stay, and increased in-hospital mortality. Therefore, it is crucial to determine the balance and identify the optimal extubation time. Despite inconsistent associations of extubation failure or prolonged intubation for certain risk factors reported in prior literatures, their exploration remains valuable. Though these factors did not show significant differences in our multivariable analysis, those found significant in univariate analysis may still be relevant and contribute to the scoring system. By integrating these variables, a more comprehensive approach for assessing extubation readiness and improving outcomes in ICH patients can be developed.

Accumulating evidence has shown that post-ICH inflammation plays an important role in secondary brain injury, which affects the clinical outcome [38, 39]. Only one study specifically examined surgical patients with ICH and found that the ICU-admission platelet-lymphocyte ratio was significantly associated with worse short-term neurological outcomes [40]. In contrast, the WBC count is reliable, easy to use, and can be routinely evaluated. Its role in ICH pathogenesis and clinical outcomes has been studied extensively [41–44]. The increase in inflammatory cells reflects the acute response to neurological injury [38]. However, studies regarding the surgical outcome of ICH have conflicting results, attributed to different surgical modalities and timing, as well as hematoma location and volume [45–48]. The current consensus on the management of ICH still states that the benefit of hematoma evacuation is not well established and regards it as a lifesaving method for those with rapid neurological deterioration or brainstem compression [49, 50].

The activated inflammatory response can also be triggered by various infections, with pneumonia being the leading cause in patients with ICH [49]. Ventilator-associated pneumonia is a common nosocomial infection, occurring in 9–28% of intubated patients after > 48 h of MV [51, 52]. It is associated with prolonged hospitalization, increased costs, morbidity, and mortality [53]. In patients under MV, the incidence of ventilator-associated pneumonia increases with the duration of ventilation. In our study, elevated WBCs were associated with extubation failure in patients with ICH after surgery. Indeed, depending solely on a single isolated laboratory value to assess the success of extubation may not be judicious. Nevertheless, we anticipate that our findings could contribute to the improvement of scoring systems, particularly those customized for neurosurgical patients who have undergone the operation. We propose that MV weaning should be delayed in the event of leukocytosis to prevent extubation failure. When patients exhibit an elevated white blood cell count during the weaning phase, it necessitates a comprehensive exploration of potentially reversible or treatable causes of leukocytosis. Addressing these underlying factors may serve to facilitate or enhance the likelihood of successful extubation.

The extubation failure rate observed in our study is 11.2% which was slightly lower than those previously reported. This outcome can be attributed, in part, to our specific focus on neurosurgical patients and the implementation of a protocolized weaning program aimed at facilitating successful liberation from the ventilator. Furthermore, in line with the recent 2022 guidelines for ICH published by the American Heart Association/American Stroke Association, which proposed the consideration of minimally invasive hematoma evacuation over conventional craniotomy for patients with supratentorial ICH exceeding 20 to 30 mL in volume and GCS scores within the moderate range (5–12) as candidates for hematoma evacuation [49], our prompt surgical intervention, with minimally invasive approach, holds promise for facilitating neurological recovery and increasing the likelihood of successful extubation.

Prolonged ventilatory support is associated with significant morbidity and mortality and is a major indication for tracheostomy [10, 33]. Increasing evidence has shown a correlation between neurological status and prolonged intubation in patients with ICH. An admission GCS of < 8 was reported to be an independent predictor of tracheostomy in nonsurgical patients with ICH [11]. Another study reported that a lower GCS on day 3 of ICH onset was associated with a higher tracheostomy rate in nonsurgical patients [54]. In line with previous studies, we demonstrated an association between low admission GCS and prolonged intubation. In addition, we showed that age is independently associated with prolonged intubation, which has never been reported in this population.

This study had some limitations. Our data were collected retrospectively from a single center. A larger population study is needed to test the generalizability of our results. Moreover, these patients were treated surgically by three neurosurgeons. Thus, the postoperative treatment strategy may vary between them. Nevertheless, this study investigates an important aspect of the treatment course in a selected patient cohort, thus providing a basis for further studies.

## Conclusion

We identified the prognostic predictors of extubation failure and prolonged MV in surgical patients with ICH. The findings can help develop risk-scoring systems and improve current weaning protocols, which is essential to improve the strategies for early initiation of adequate treatment and prognosis assessment.

## Data Availability

The datasets generated during and/or analyzed during the current study are available from the corresponding author upon reasonable request.

## List of abbreviations

ICH: Intracerebral hemorrhage.
MV: Mechanical ventilation.
GCS: Glasgow Coma Scale
ICU: Intensive care unit
SBT: Spontaneous breathing trials
CT: Computed tomography
ICP: Intracranial pressure.
PiMax: Maximal inspiratory pressure
PeMax: Maximum expiratory pressure.
RR: Respiratory rate
RSBI: Rapid shallow breathing index
VE: Minute ventilation
VT: Tidal volume
WBC: White blood cell
